# Nutritional risk, inflammation, and hospitalization after hemodialysis initiation

**DOI:** 10.1080/0886022X.2026.2757931

**Published:** 2026-11-11

**Authors:** Hikaru Uematsu, Tatsunori Toida, Yuri Tanaka, Nobuhiko Joki

**Affiliations:** Division of Nephrology, Toho University Ohashi Medical Center, Tokyo, Japan

**Keywords:** C-reactive protein, Geriatric Nutritional Risk Index, hemodialysis initiation, length of stay, prolonged post-initiation stay

## Abstract

Evidence linking nutritional and inflammatory markers at hemodialysis initiation to subsequent hospital stay remains limited. We examined whether the Geriatric Nutritional Risk Index (GNRI) and C-reactive protein (CRP) at hemodialysis initiation were associated with prolonged post-initiation hospital stay. This single-center retrospective study included patients who initiated hemodialysis during hospitalization and transitioned to maintenance hemodialysis between 2004 and 2024. The primary outcome was hospitalization for 30 days or longer after hemodialysis initiation. Multivariable logistic regression was used with adjustment for age, sex, cardiovascular disease, diabetes mellitus, initiation modality, calendar period, and pre-hemodialysis vascular access status. Among 517 patients, 501 were included in multivariable analysis, and 166 patients (32.1%) experienced prolonged post-initiation stay. A higher GNRI was associated with lower odds of prolonged stay (odds ratio per 1-unit increase, 0.955; 95% confidence interval, 0.925–0.986; *p* = 0.005), whereas higher log-transformed CRP was associated with higher odds (odds ratio, 1.203; 95% confidence interval, 1.055–1.372; *p* = 0.006). The low GNRI/high CRP group had the highest odds of prolonged stay (odds ratio, 3.180; 95% confidence interval, 1.489–6.787; *p* = 0.003), without significant multiplicative interaction. Lower GNRI and higher CRP at hemodialysis initiation were independently and jointly associated with prolonged post-initiation stay. These findings describe associations rather than validated predictions, and prospective evaluation is required before these markers are applied to individual-level risk assessment.

## LAY SUMMARY

Some people who start hemodialysis in hospital need a prolonged stay afterwards. We reviewed records of 517 patients who started hemodialysis during hospitalization at one hospital in Tokyo, Japan, between 2004 and 2024. We examined two measures obtained just before the first hemodialysis session: a nutrition-related score based on blood albumin and body weight (Geriatric Nutritional Risk Index, GNRI) and a blood marker of inflammation (C-reactive protein, CRP). Lower GNRI and higher CRP were associated with staying in hospital for 30 days or longer after starting hemodialysis. Patients with both low GNRI and high CRP were more likely to have a prolonged stay than those with neither. This study of past records shows associations rather than cause and effect. It did not establish whether these measures can predict hospital stay for individual patients.

## KEY MESSAGES

**What is known:** Malnutrition and inflammation frequently coexist at hemodialysis initiation and predict mortality, but their relationship with the duration of the initiation hospitalization itself has rarely been examined.

**This study adds:** Among 517 patients initiating maintenance hemodialysis during hospitalization over 20 years, lower GNRI and higher CRP immediately before the first session were independently and jointly associated with prolonged post-initiation stay (≥30 days), with broadly consistent directions across alternative outcome definitions and modeling strategies.

**Potential impact:** Two routinely available markers may help characterize illness burden during hemodialysis initiation.

## GRAPHICAL ABSTRACT

**Figure:**
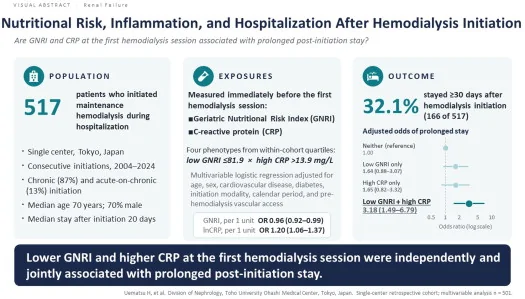

## Introduction

The transition from end-stage kidney disease to hemodialysis initiation is a clinically vulnerable period for patients. During this phase, multiple factors, including uremia, fluid overload, infection, heart failure, and vascular access-related procedures, may contribute to prolonged hospitalization after dialysis initiation [1,2]. Length of hospital stay is therefore an important clinical outcome, reflecting not only patient burden but also healthcare resource use [3].

Hospitalization-associated disability is a recognized consequence of hospitalization [4]. Among incident dialysis patients, frailty is common and is associated with subsequent hospitalization [5], and longer hospital stay has been linked to decline in activities of daily living [6]. Thus, prolonged hospitalization during dialysis initiation has particular clinical significance.

The clinical course after hemodialysis initiation is also influenced by initiation modality and the degree of pre-hemodialysis vascular access preparation, both of which have been associated with greater burden at initiation and worse outcomes [7–9]. However, even after accounting for these background factors, evidence remains limited regarding how nutritional status and inflammatory burden at the time of hemodialysis initiation affect the subsequent in-hospital course.

The Geriatric Nutritional Risk Index (GNRI) is a simple, objective nutrition-related index calculated from serum albumin and body weight [10,11], and it has been widely investigated as a prognostic indicator of all-cause and cardiovascular mortality in patients on maintenance hemodialysis [12]. Even in the hemodialysis initiation setting, a low GNRI has been associated with higher cardiovascular mortality in retrospective cohorts, although a causal relationship remains to be established [13]. Meanwhile, C-reactive protein (CRP) is a well-established marker reflecting chronic inflammation and acute exacerbation in chronic kidney disease (CKD) and dialysis populations, with documented links to all-cause [14] and cardiovascular mortality [15]. In contrast, studies directly examining its relationship with length of hospital stay remain scarce. Because the GNRI is derived from serum albumin and body weight, it may be influenced by fluid retention and inflammation, which limits its specificity as a standalone nutritional marker [16]. In CKD and dialysis populations, the interplay between malnutrition and inflammation is recognized as the malnutrition–inflammation complex syndrome, supporting the potential value of combined assessment at hemodialysis initiation [17].

With regard to hospitalization, Pupim et al. [18] reported that nutritional parameters at the initiation of maintenance hemodialysis, including serum albumin and serum creatinine, were associated with hospital admissions and length of stay during the first year of therapy, and Uludag et al. [19] reported that lower serum albumin was associated with a higher risk of hospital admission and longer hospital stay among incident hemodialysis patients. Both studies addressed hospitalization occurring after the initiation period. In contrast, the duration of the hospitalization during which hemodialysis is initiated, a period with direct implications for patients and healthcare systems, has rarely been examined, and whether a combined assessment of nutritional risk and inflammation at the first session is associated with it remains unclear.

To address these gaps, we retrospectively examined the associations of GNRI and CRP at hemodialysis initiation with prolonged post-initiation stay, including an exploratory four-phenotype analysis.

## Materials and methods

### Study design and participants

This single-center retrospective observational study included consecutive patients who initiated hemodialysis at Toho University Ohashi Medical Center between January 2004 and December 2024. Clinical characteristics at hemodialysis initiation, laboratory data, and hospitalization course were retrieved from electronic medical records to construct a hemodialysis initiation database. From this cohort, we included patients who initiated hemodialysis during the course of chronic kidney disease and subsequently transitioned to maintenance hemodialysis, corresponding to the chronic and acute-on-chronic (AoC) groups. Chronic initiation was operationally defined as initiation of maintenance hemodialysis resulting from progression of preexisting chronic kidney disease without a discrete acute deteriorating event, and AoC initiation as initiation triggered by an acute deterioration superimposed on preexisting chronic kidney disease, followed by transition to maintenance hemodialysis. This classification was based on the clinical judgment of the attending nephrologists at initiation and represents a study-specific operational definition, conceptually informed by the framework of acute kidney injury and acute kidney disease superimposed on chronic kidney disease [20]; no universally standardized definition of AoC initiation of maintenance hemodialysis has been established. This classification is distinct from planned versus unplanned initiation, which was not directly recorded in our database. We excluded patients with acute hemodialysis initiation primarily due to acute kidney injury (acute group), those with unknown initiation modality, and those who initiated hemodialysis in the outpatient setting. Patients without a confirmed discharge date were also excluded.

At our center, initial hemodialysis prescriptions generally followed an institutional approach, starting with a shorter treatment time and lower blood flow rate (three sessions per week, 3 h per session) that were gradually increased according to clinical status, although prescriptions were individualized in some patients. Session-level prescription data, including treatment time, blood flow rate, and dialyzer membrane area, were not recorded in the database.

The study protocol was approved by the Ethics Committee of Toho University School of Medicine (Approval Number: T2025-100), and the study was conducted in accordance with the principles of the Declaration of Helsinki. Because of the retrospective observational design using existing medical records, the requirement for written informed consent was waived by the Ethics Committee, and an opt-out approach was used to provide participants the opportunity to decline participation, in accordance with the policy of Toho University School of Medicine and the Japanese Ethical Guidelines for Medical and Health Research Involving Human Subjects.

### Primary exposures

The primary exposures were GNRI and CRP. Laboratory values at hemodialysis initiation, including serum albumin and CRP, were the most recent measurements obtained immediately before the first hemodialysis session. GNRI was calculated using the Bouillanne formula: 14.89 × serum albumin (g/dL) + 41.7 × (actual body weight/ideal body weight) [10]. Ideal body weight (IBW) was derived using a body mass index of 22 kg/m^2^. When actual body weight exceeded IBW, the body weight/IBW ratio was capped at 1. Body weight at hemodialysis initiation was used preferentially; when unavailable, admission body weight was substituted (6 patients, 1.1%). Owing to its skewed distribution, CRP was analyzed as a continuous variable after natural logarithmic transformation (lnCRP) using ln(CRP + 0.1), with CRP expressed in mg/L.

For the exploratory four-phenotype analysis, low GNRI was defined as the first quartile of GNRI (≤81.9) and high CRP as the highest quartile of CRP within the main analysis cohort (>13.9 mg/L [1.39 mg/dL]). These thresholds were derived from within-cohort quartiles and are therefore sample-derived; they are not proposed as externally validated clinical cutoffs. Patients were classified into four groups: non-low GNRI/non-high CRP, low GNRI only, high CRP only, and low GNRI/high CRP. In addition, exploratory analyses based on the malnutrition–inflammation–atherosclerosis (MIA) concept were performed, defining malnutrition as GNRI <92, inflammation as CRP >3 mg/L, and atherosclerosis approximated by a history of cardiovascular disease [10,21–23]. The MIA score (0–3), a malnutrition–inflammation score (0–2) excluding the atherosclerosis component, and eight MIA-related phenotype groups were derived from these components.

### Covariates

Covariates included age, sex, history of cardiovascular disease, diabetes mellitus, initiation modality (chronic versus AoC), pre-hemodialysis vascular access status, and calendar period (2004–2008, 2009–2013, 2014–2018, and 2019–2024). History of cardiovascular disease was defined as the presence of ischemic heart disease, heart failure, cerebrovascular disease, or peripheral artery disease. Pre-hemodialysis vascular access was defined as vascular access for maintenance hemodialysis, including arteriovenous fistula, arteriovenous graft, superficialized artery, or long-term hemodialysis catheter, that had been created or placed on or before the date of the first hemodialysis session. In patients with an unrecorded access creation date, those whose medical records clearly confirmed the absence of vascular access at initiation were classified as having no pre-hemodialysis access. Patients who could not be definitively classified into either category were treated as having missing data for this key covariate and were excluded from the multivariable analyses. Covariates were selected on the basis of clinical relevance during the hemodialysis initiation period and previously reported associations with hospitalization; no data-driven variable selection was performed.

### Outcomes

The primary outcome was prolonged post-initiation stay, defined as a hospital stay of ≥30 days from hemodialysis initiation to discharge. In this cohort, the median length of stay was 20 days, and 30 days corresponded to the upper quartile of the chronic group. This threshold was also considered clinically interpretable in light of prior literature linking longer hospital stay to functional decline in hemodialysis patients [6]. The secondary outcome was length of hospital stay after hemodialysis initiation, analyzed as a natural log-transformed continuous variable. Patients who died during the index hospitalization were classified according to their observed length of stay in the primary analysis, and a composite outcome of prolonged post-initiation stay or in-hospital death was examined in a sensitivity analysis.

### Statistical analysis

Baseline characteristics were compared across phenotype groups using the Kruskal–Wallis test for continuous variables and the chi-square test for categorical variables. Multivariable logistic regression analysis was performed for the primary outcome, with results presented as odds ratios (ORs) and 95% confidence intervals (CIs). Because length of hospital stay after initiation takes only positive values and was markedly right-skewed, it was natural log-transformed and analyzed using multivariable linear regression. Robust standard errors (sandwich estimator) were applied across all regression models.

The multiplicative interaction between GNRI and lnCRP was evaluated by including an interaction term (GNRI × lnCRP) in the model. Multicollinearity between GNRI and lnCRP was assessed using variance inflation factors.

For the log-linear model, residual skewness and kurtosis, the Breusch–Pagan test, and the Shapiro–Wilk test were examined. Because length of stay was overdispersed relative to a Poisson distribution, negative binomial regression and a gamma generalized linear model with a log link were also fitted. Time to discharge alive was analyzed using Cox regression and Fine–Gray models treating in-hospital death as a competing event. In the complete-case sample, apparent discrimination was assessed using the area under the receiver operating characteristic curve (AUC) and compared using the DeLong test; because AUCs were estimated in the model-fitting sample and the DeLong test may be conservative for nested models, these comparisons were considered exploratory. Model fit was compared using the Akaike information criterion (AIC), and descriptive integrated discrimination improvement (IDI) values were calculated. We also performed an exploratory analysis stratified by whether the weight component of GNRI was capped.

As an additional exploratory analysis, the MIA score (number of components present, 0–3), the malnutrition–inflammation score (0–2), and eight phenotype groups defined by the presence or absence of the three components were each entered in separate multivariable logistic regression models in place of GNRI and lnCRP, with score 0 or the group without any component as the reference; the MIA score was also analyzed per 1-point increase. Because history of cardiovascular disease defined the atherosclerosis component, it was not included as a covariate in these models. Sensitivity analyses comprised (1) alternative length-of-stay cutoffs (≥14, ≥21, ≥45, and ≥60 days, and the 75th percentile of the main analysis cohort, ≥34 days); (2) a composite outcome of prolonged post-initiation stay or in-hospital death; (3) restriction to chronic initiation, to patients who survived to discharge, and to patients initiating hemodialysis in 2014–2024; (4) stratification by pre-hemodialysis vascular access status, with interaction terms; (5) additional adjustment for pulmonary edema, white blood cell (WBC) count, comorbidities (malignancy, autoimmune disease, and respiratory disease), and primary kidney disease; and (6) the alternative outcome models described above. Results of these sensitivity analyses are presented in Supplementary Tables S2–S5, and the distributions of initiation modality, pre-hemodialysis vascular access status, and primary kidney disease by calendar period are presented in Supplementary Table S6. Both the four-phenotype and MIA-related analyses were considered exploratory. All statistical analyses were performed using Stata/MP version 19.0 (StataCorp, College Station, TX, USA). A two-sided *p*-value < 0.05 was considered statistically significant.

**Table 1.** Baseline characteristics of the main analysis cohort according to GNRI/CRP phenotype.

Variable	Overall (*n* = 517)	Non-low GNRI/non-high CRP (*n* = 308)	Low GNRI only (*n* = 80)	High CRP only (*n* = 75)	Low GNRI + high CRP (*n* = 54)	*p*-value
Patient characteristics and comorbidities						
Age, years	70 [58–79]	69 [56.5–77]	69.5 [58.5–80]	71 [60–79]	77 [66–83]	<0.001
Body weight, kg	61.5 [51.8–69.9]	62.1 [51.8–70.6]	60.1 [47.7–69.4]	62.2 [54.3–72.0]	59.7 [53.0–67.6]	0.430
Male sex, *n* (%)	360 (69.6)	214 (69.5)	52 (65.0)	56 (74.7)	38 (70.4)	0.631
Diabetes mellitus, *n* (%)	269 (52.0)	163 (52.9)	46 (57.5)	36 (48.0)	24 (44.4)	0.425
History of CVD, *n* (%)	227 (43.9)	128 (41.6)	41 (51.3)	36 (48.0)	22 (40.7)	0.366
Laboratory values						
GNRI	87.9 [81.9–93.6]	90.8 [86.4–95.2]	78.9 [75.6–80.4]	89.3 [86.4–93.8]	74.3 [71.5–80.3]	<0.001
CRP, mg/L	3.1 [0.7–13.9]	1.0 [0.4–4.0]	2.4 [1.0–5.2]	39.0 [24.2–70.2]	41.4 [21.8–93.6]	<0.001
Albumin, g/dL	3.2 [2.8–3.5]	3.4 [3.1–3.7]	2.6 [2.4–2.7]	3.3 [3.1–3.6]	2.3 [2.0–2.6]	<0.001
Creatinine, mg/dL	8.92 [7.10–11.4]	9.28 [7.55–11.8]	8.29 [6.55–10.6]	8.89 [6.96–11.6]	7.61 [6.00–9.76]	<0.001
WBC, /μL	6,050 [4,900–7,600]	5,800 [4,700–7,000]	6,150 [4,900–7,400]	7,400 [5,500–9,400]	8,150 [5,700–9,600]	<0.001
Initiation characteristics						
AoC, *n* (%)	68 (13.2)	30 (9.7)	10 (12.5)	16 (21.3)	12 (22.2)	0.010
Pre-HD access present, *n* (%)^a^	383/501 (76.4)	260/300 (86.7)	54/78 (69.2)	41/70 (58.6)	28/53 (52.8)	<0.001
DL catheter, *n* (%)^a^	20/501 (4.0)	6/302 (2.0)	3/77 (3.9)	5/71 (7.0)	6/51 (11.8)	0.005
Pulmonary edema, *n* (%)^b^	133/491 (27.1)	53/296 (17.9)	23/76 (30.3)	31/68 (45.6)	26/51 (51.0)	<0.001
Calendar period, *n* (%)						<0.001
2004–2008	118 (22.8)	75 (24.4)	20 (25.0)	21 (28.0)	2 (3.7)	
2009–2013	135 (26.1)	92 (29.9)	11 (13.8)	26 (34.7)	6 (11.1)	
2014–2018	134 (25.9)	67 (21.8)	30 (37.5)	14 (18.7)	23 (42.6)	
2019–2024	130 (25.1)	74 (24.0)	19 (23.8)	14 (18.7)	23 (42.6)	

Continuous variables are presented as median [interquartile range], and categorical variables as *n* (%). Conversion factor for CRP: mg/L to mg/dL, ×0.1.

^a^Pre-HD access and DL catheter data were each available in 501 patients overall; because missingness patterns differed between these variables, phenotype-specific denominators are shown separately.

^b^Pulmonary edema was available in 491 patients. WBC was available in 508 patients. AoC, acute-on-chronic; CRP, C-reactive protein; CVD, cardiovascular disease; DL, double-lumen catheter; GNRI, Geriatric Nutritional Risk Index; HD, hemodialysis; IQR, interquartile range; WBC, white blood cell count.

**Table 2.** Multivariable associations of GNRI and lnCRP with post-initiation hospitalization outcomes (*n* = 501).

	Prolonged stay ≥30 days		Log-transformed hospitalization days	
Variable	OR (95% CI)	*p*-value	*β* (95% CI)	*p*-value
GNRI (per 1-unit increase)	0.955 (0.925–0.986)	0.005	−0.016 (−0.024 to −0.009)	<0.001
lnCRP (per 1-unit increase)	1.203 (1.055–1.372)	0.006	0.051 (0.020–0.082)	0.001
Age (per year)	1.024 (1.005–1.044)	0.015	0.008 (0.003–0.012)	0.001
Male	0.505 (0.312–0.817)	0.005	−0.145 (−0.270 to −0.020)	0.023
History of cardiovascular disease	0.999 (0.621–1.604)	0.995	0.055 (−0.061 to 0.170)	0.352
Diabetes mellitus	1.144 (0.704–1.859)	0.588	0.004 (−0.114 to 0.123)	0.941
AoC initiation (vs. chronic)	2.325 (1.151–4.697)	0.019	0.177 (0.001–0.354)	0.049
Calendar period 2009–2013 (vs. 2004–2008)	0.690 (0.363–1.310)	0.257	−0.127 (−0.298 to 0.044)	0.146
Calendar period 2014–2018 (vs. 2004–2008)	0.421 (0.212–0.835)	0.013	−0.496 (−0.689 to −0.304)	<0.001
Calendar period 2019–2024 (vs. 2004–2008)	0.181 (0.088–0.374)	<0.001	−0.584 (−0.773 to −0.395)	<0.001
Pre-hemodialysis vascular access	0.205 (0.118–0.356)	<0.001	−0.633 (−0.779 to −0.488)	<0.001

Multivariable analyses were performed in 501 patients. Models were adjusted for age, sex, history of cardiovascular disease, diabetes mellitus, initiation modality (chronic vs. AoC), calendar period, and pre-HD access status, using robust standard errors. Reference categories were female sex, no history of cardiovascular disease, no diabetes mellitus, chronic initiation, calendar period 2004–2008, and no pre-hemodialysis vascular access. ORs are per 1-unit increase in GNRI and per 1-unit increase in lnCRP; *β* coefficients represent the change in ln-transformed hospitalization days per 1-unit increase. AoC, acute-on-chronic; GNRI, Geriatric Nutritional Risk Index; HD, hemodialysis; lnCRP, natural log-transformed C-reactive protein; OR, odds ratio.

## Results

### Baseline characteristics and study cohorts

Of the 620 patients who initiated hemodialysis during the study period, 36 were excluded because of acute initiation or unknown initiation modality, leaving 584 patients in the at-risk cohort. A further 24 patients who initiated hemodialysis in the outpatient setting and 43 patients with missing data required for multivariable modeling (GNRI unavailable because of missing serum albumin or body weight, *n* = 32; CRP unavailable, *n* = 2; or missing history of cardiovascular disease, *n* = 9) were excluded, resulting in 517 patients in the main analysis cohort. The multivariable primary analysis included 501 patients after exclusion of 16 with indeterminate pre-hemodialysis vascular access status (Figure 1).

**Figure 1.**
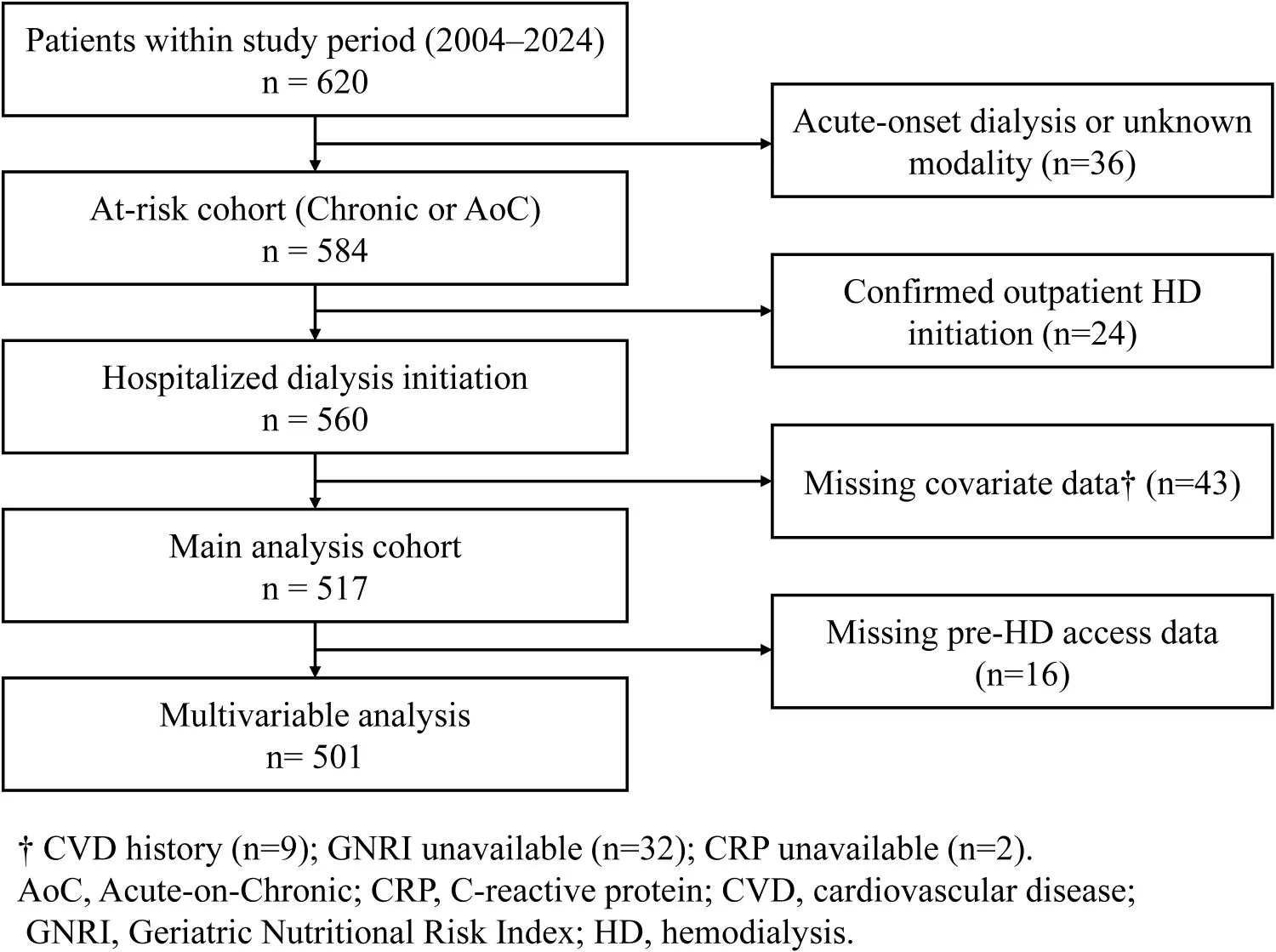
Patient enrollment and exclusion flow diagram. Flow diagram showing the selection of the at-risk cohort, main analysis cohort, and multivariable primary analysis cohort.

Baseline characteristics of the main analysis cohort are summarized in Table 1. The low GNRI/high CRP group accounted for 10.4% of the main analysis cohort (54 of 517 patients; 53 in the multivariable analysis cohort). Significant differences across the four phenotype groups were observed in age, GNRI, CRP, albumin, WBC count, initiation modality, pre-hemodialysis vascular access status, double-lumen catheter use, pulmonary edema, and calendar period, whereas sex, diabetes mellitus, and history of cardiovascular disease did not differ significantly. Median (75th percentile) length of stay after initiation was 18 (30) days for chronic and 40 (63) days for AoC initiation, and 17 (27) days in patients with, versus 37.5 (61) days in patients without, pre-hemodialysis vascular access. Pulmonary edema at initiation was more frequent among patients whose actual-to-ideal body-weight ratio was capped at 1 than among those whose ratio was not capped (99/318 [31.1%] vs. 34/173 [19.7%], *p* = 0.006).

### Associations with prolonged post-initiation stay

Of the 517 patients in the main analysis cohort, 166 (32.1%) experienced prolonged post-initiation stay. Multivariable logistic regression analysis (*n* = 501) showed that each 1-unit increase in GNRI was independently associated with lower odds of prolonged post-initiation stay (odds ratio [OR], 0.955; 95% confidence interval [CI], 0.925–0.986; *p* = 0.005), whereas each 1-unit increase in lnCRP was independently associated with higher odds (OR, 1.203; 95% CI, 1.055–1.372; *p* = 0.006). Other factors independently associated with prolonged post-initiation stay included AoC initiation versus chronic initiation (OR, 2.325; 95% CI, 1.151–4.697; *p* = 0.019), the presence of pre-hemodialysis vascular access (OR, 0.205; 95% CI, 0.118–0.356; *p* < 0.001), age (OR, 1.024; *p* = 0.015), and male sex (OR, 0.505; *p* = 0.005). No significant associations were observed for history of cardiovascular disease or diabetes mellitus. Consistent findings were obtained when log-transformed length of hospital stay was analyzed as a continuous outcome (GNRI: *β* = −0.016, *p* < 0.001; lnCRP: *β* = 0.051, *p* = 0.001) (Table 2).

### Analysis of four-group phenotypes

The four phenotype groups were defined within the main analysis cohort (*n* = 517), and multivariable analyses were conducted among the 501 patients with available pre-hemodialysis vascular access data (Table 3). Compared with the reference group (non-low GNRI/non-high CRP, *n* = 300), neither the low GNRI-only group (*n* = 78) nor the high CRP-only group (*n* = 70) was significantly associated with prolonged post-initiation stay after multivariable adjustment. In contrast, the low GNRI/high CRP group (*n* = 53) showed the highest odds (OR, 3.180; 95% CI, 1.489–6.787; *p* = 0.003), and this group also showed the strongest association in the analysis of log-transformed length of hospital stay as a continuous outcome (*β* = 0.441; *p* < 0.001). In a model including the GNRI × lnCRP interaction term, no significant multiplicative interaction was observed (OR, 0.998; *p* = 0.765).

**Table 3.** Exploratory analysis of combined GNRI/CRP phenotypes and post-initiation hospitalization outcomes.

	Prolonged stay ≥30 days		Log-transformed hospitalization days	
Phenotype group vs. reference^a^	OR (95% CI)	*p*-value	*β* (95% CI)	*p*-value
Low GNRI only	1.640 (0.876–3.072)	0.122	0.189 (−0.005 to 0.382)	0.056
High CRP only	1.648 (0.819–3.316)	0.161	0.161 (−0.032 to 0.354)	0.101
Low GNRI + high CRP	3.180 (1.489–6.787)	0.003	0.441 (0.229–0.654)	<0.001

Multivariable analyses were performed in 501 patients. Phenotype thresholds were derived within the main analysis cohort: low GNRI ≤81.9 (first quartile) and high CRP >13.9 mg/L (1.39 mg/dL; above the 75th percentile). These are sample-derived thresholds and have not been externally validated. lnCRP was used as a continuous variable in the main regression models (Table 2), whereas phenotype groups were defined using the original CRP scale. Conversion factor for CRP: mg/L to mg/dL, ×0.1.

^a^Reference group: Non-low GNRI/Non-high CRP. Models were adjusted for the same covariates as in the main analysis. AoC, acute-on-chronic; CRP, C-reactive protein; GNRI, Geriatric Nutritional Risk Index; lnCRP, natural log-transformed C-reactive protein; OR, odds ratio.

### Sensitivity analyses

The directions of the main associations were broadly consistent across sensitivity analyses, although the precision of individual estimates varied. Estimates were similar when the cohort was restricted to chronic initiation (*n* = 437) or to patients who survived to discharge (*n* = 493) (Supplementary Table S2), and after additional adjustment for pulmonary edema (*n* = 487), comorbidities (*n* = 500), and primary kidney disease (*n* = 501) (Supplementary Table S5). Nine patients died during the index hospitalization (median length of stay, 66 days); seven had stays of ≥30 days and two died before day 30. With a composite outcome of prolonged post-initiation stay or in-hospital death (168 events), estimates were essentially unchanged (GNRI: OR, 0.954; lnCRP: OR, 1.218) (Supplementary Table S4).

Among patients with pre-hemodialysis vascular access (*n* = 383), higher GNRI remained associated with lower odds of prolonged stay (OR, 0.961; 95% CI, 0.924–0.998; *p* = 0.042), and higher lnCRP with higher odds (OR, 1.255; 95% CI, 1.071–1.471; *p* = 0.005). In this stratum, the low GNRI/high CRP group had the highest odds among the four phenotypes (OR vs. the non-low GNRI/non-high CRP group, 4.757; 95% CI, 1.803–12.554; *p* = 0.002). Tests for interaction between access status and GNRI (*p* = 0.304) or lnCRP (*p* = 0.408) were not significant (Supplementary Table S2).

After additional adjustment for WBC count within the same analytic sample (*n* = 500), the association of lnCRP was attenuated and no longer statistically significant (OR, 1.136; 95% CI, 0.989–1.304; *p* = 0.072), whereas the estimate for GNRI was essentially unchanged (OR, 0.956; *p* = 0.006) (Supplementary Table S5). When the cohort was restricted to patients initiating hemodialysis in 2014–2024 (*n* = 263), the association of lnCRP persisted (OR, 1.378; 95% CI, 1.103–1.723; *p* = 0.005), whereas the estimate for GNRI was attenuated and imprecise (OR, 0.978; 95% CI, 0.932–1.027; *p* = 0.369), with a confidence interval compatible with both the full-cohort estimate and no association (Supplementary Table S2). Across calendar periods, the proportion of AoC initiation declined (*p* < 0.001), whereas the proportion of patients with pre-hemodialysis vascular access (*p* = 0.086) and the distribution of primary kidney disease (*p* = 0.058) did not differ significantly (Supplementary Table S6).

Residuals of the log-linear model were approximately symmetric (skewness 0.31) with mildly heavy tails (kurtosis 4.54; 3 for a normal distribution), and the Breusch–Pagan test showed no significant heteroskedasticity (*p* = 0.132), although the Shapiro–Wilk test indicated departure from normality (*W* = 0.984, *p* < 0.001). Given marked overdispersion of length of stay (mean 29.6 days; variance 1575.6), negative binomial regression (incidence rate ratio [IRR], 0.979 for GNRI and 1.072 for lnCRP) and a gamma generalized linear model yielded estimates consistent with the log-linear model. In time-to-discharge analyses (493 discharges among 501 patients), higher GNRI was associated with earlier discharge alive and higher lnCRP with delayed discharge in Cox models (hazard ratio [HR], 1.027 and 0.897) and in Fine–Gray models treating in-hospital death as a competing event (subdistribution HR, 1.025 and 0.890; all *p* < 0.001) (Supplementary Table S4). Estimates were directionally consistent across alternative length-of-stay cutoffs, although the 95% CI included the null for GNRI at the 21-day cutoff (*p* = 0.053) and for lnCRP at the 34-day cutoff (*p* = 0.110; 131 events) (Supplementary Table S3).

### Discrimination and model fit

Within the complete-case sample (*n* = 501), the AUC was 0.792 for the model including clinical covariates alone, 0.803 with the addition of serum albumin, 0.813 with albumin and lnCRP, 0.814 with GNRI and lnCRP, and 0.813 with albumin, body mass index, and lnCRP. The apparent AUC increased numerically with the addition of GNRI and lnCRP to the clinical-covariate model (0.792 to 0.814; exploratory DeLong *p* = 0.073). Apparent AUCs were similar for the models including GNRI and lnCRP versus albumin and lnCRP (0.814 vs. 0.813; exploratory DeLong *p* = 0.881), and the AIC was slightly lower for the albumin-based model (500.5 vs. 501.6). Descriptive IDI values were +0.034 for the addition of GNRI and lnCRP to clinical covariates and −0.001 for GNRI and lnCRP relative to albumin and lnCRP; no formal inference was performed for these point estimates.

In 339 of 517 patients (65.6%), the actual-to-ideal body-weight ratio exceeded 1 and was capped, rendering GNRI a linear transformation of serum albumin within this subgroup (*r* = 1.000); the overall correlation between GNRI and albumin was 0.949. In an exploratory analysis restricted to patients whose weight component was not capped (*n* = 174), the point estimate for GNRI was farther from the null than in the overall cohort (OR, 0.918; 95% CI, 0.871–0.968; *p* = 0.001), although effect modification by capping status was not formally tested (Supplementary Table S2).

### Additional exploratory analyses

In exploratory analyses based on the MIA framework, the malnutrition–inflammation score was associated with prolonged post-initiation stay (Figure 2). Compared with score 0, the adjusted OR was 1.225 (95% CI, 0.587–2.556; *p* = 0.589) for score 1 and 3.781 (95% CI, 1.807–7.913; *p* < 0.001) for score 2. Results for the full MIA score and the eight phenotype groups are provided in Supplementary Table S1.

**Figure 2.**
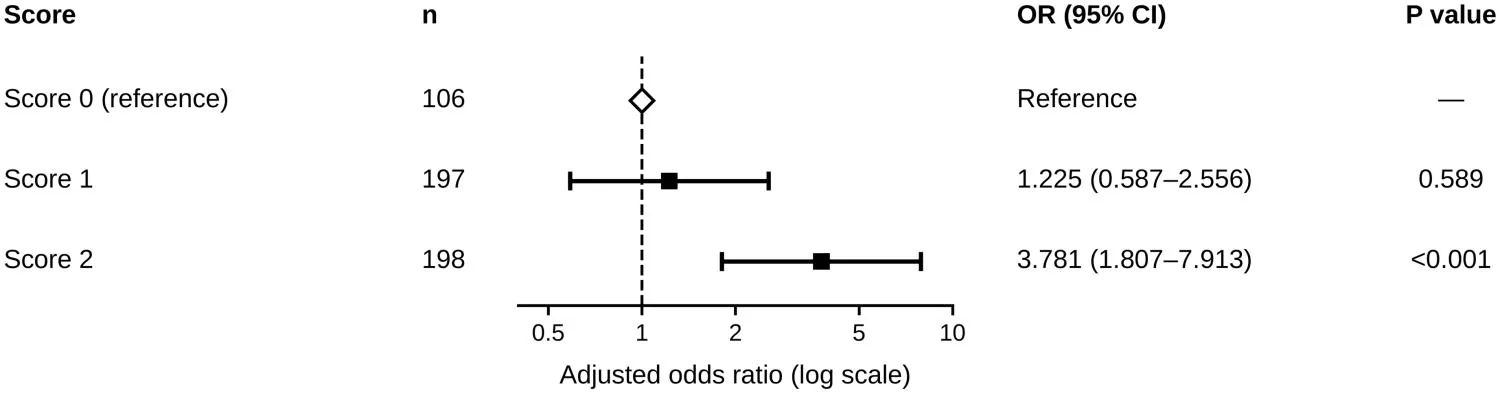
Association between malnutrition–inflammation score and prolonged post-initiation stay. Malnutrition was defined as GNRI <92 and inflammation as CRP >3 mg/L; the malnutrition–inflammation score (0–2) was the number of these components present. Adjusted odds ratios and 95% confidence intervals were estimated using multivariable logistic regression with adjustment for age, sex, diabetes mellitus, initiation modality, calendar period, and pre-hemodialysis vascular access status; history of cardiovascular disease was not included, consistent with the MIA-related analyses (Supplementary Table S1). Score 0 was used as the reference category. Group sizes within the multivariable analysis cohort (*n* = 501) were 106, 197, and 198 for scores 0, 1, and 2, respectively. CI, confidence interval; CRP, C-reactive protein; GNRI, Geriatric Nutritional Risk Index; MIA, malnutrition–inflammation–atherosclerosis; OR, odds ratio.

## Discussion

In the present study, low GNRI and elevated CRP at hemodialysis initiation were each independently associated with prolonged post-initiation hospital stay. These associations remained after adjustment for clinical background factors, including initiation modality and pre-hemodialysis vascular access status, and similar findings were observed when length of hospital stay was analyzed as a continuous outcome. In addition, the exploratory four-phenotype analysis showed that the low GNRI/high CRP group had the highest odds of prolonged post-initiation stay. These findings suggest that nutritional risk and inflammatory burden at hemodialysis initiation are associated with the subsequent in-hospital course.

Previous studies have shown that GNRI is associated with mortality-related outcomes in maintenance and incident hemodialysis patients [12,13,24,25]. Our study extends these findings by focusing on length of stay, a clinically relevant outcome in routine practice. Prolonged hospitalization may worsen functional decline during dialysis initiation, particularly in patients with frailty [5,26–28]. Accordingly, characterizing factors associated with prolonged post-initiation stay may help describe patients with a more complicated initiation course, although whether such characterization can guide intervention requires prospective evaluation. However, given the retrospective observational design, the observed associations cannot be interpreted as evidence of causality.

CRP functions as a marker of inflammatory burden in patients with advanced CKD and those undergoing hemodialysis, and its elevation has been associated with all-cause and cardiovascular mortality [14,15]. Furthermore, CRP is considered a prognostic indicator that is associated with clinical outcomes independently of serum albumin and other confounding factors [29]. At the time of hemodialysis initiation, multiple clinical conditions, including uremia, fluid overload, heart failure, and infection, frequently coexist. CRP levels may therefore nonspecifically reflect the overall severity of these coexisting pathologies. In our cohort, a higher CRP level was independently associated with prolonged post-initiation stay even after adjustment for GNRI, suggesting that inflammatory status may provide information complementary to nutritional assessment. After additional adjustment for WBC count, however, the association of lnCRP was attenuated (*p* = 0.072), whereas the estimate for GNRI was essentially unchanged. This pattern suggests that the two markers capture partly distinct aspects of illness at initiation, with CRP more closely tied to acute inflammatory activity or illness severity; CRP at initiation should therefore not be over-interpreted as a specific marker of chronic inflammatory burden.

Collinearity between GNRI and CRP was low in our models: variance inflation factors remained low, and the adjusted odds ratio for GNRI changed little when lnCRP was omitted (0.947 vs. 0.955). However, GNRI should not be interpreted as a specific measure of malnutrition in this setting. Because GNRI incorporates serum albumin, it is partly influenced by inflammation-related hypoalbuminemia [30], and its body-weight component is sensitive to fluid status at hemodialysis initiation: in about two-thirds of our patients the actual-to-ideal weight ratio was capped at 1, rendering GNRI a linear transformation of serum albumin, and pulmonary edema at initiation was more frequent among these patients. Consistent with this, GNRI was highly correlated with albumin, and models including GNRI or albumin had similar apparent AUCs, with the AIC marginally favoring the albumin-based model. GNRI in this population may therefore be better understood as a composite marker influenced by nutritional status, inflammation-related hypoalbuminemia, volume status, and overall illness severity, rather than as a nutrition-specific index. The apparent contrast with the incremental prognostic value of GNRI for cardiovascular mortality reported by Takahashi et al. [13] may relate to the different outcomes, namely long-term cardiovascular mortality versus the length of a single initiation hospitalization, and to differences in the timing and definition of the body weight used to calculate GNRI, although this cannot be verified from our data.

In the four-phenotype analysis, the low GNRI/high CRP group showed the highest odds of prolonged post-initiation stay (OR, 3.180). This finding is consistent with previous reports suggesting that the coexistence of malnutrition and inflammatory burden is associated with adverse outcomes [31,32]. However, those studies differed from the present study in terms of patient populations and clinical outcomes, and evidence directly evaluating the combination of GNRI and CRP during the hemodialysis initiation period remains limited. Therefore, these results should be interpreted as exploratory. Furthermore, the multiplicative interaction between GNRI and lnCRP was not statistically significant (*p* = 0.765). We describe the phenotype findings as a joint association of the two markers and do not interpret them as evidence of biological synergy. In the exploratory eight-phenotype analysis, phenotypes combining low GNRI and elevated CRP tended to show higher odds; however, these findings should be interpreted cautiously given the small subgroup sizes and the approximation of atherosclerosis by cardiovascular disease history. A history of cardiovascular disease was not associated with prolonged post-initiation stay in this cohort, and the malnutrition–inflammation score without the atherosclerosis component showed a broadly similar pattern of point estimates to the full MIA score. We therefore frame the present findings within a malnutrition–inflammation relationship rather than the full MIA syndrome, while noting that these comparisons did not formally test the incremental contribution of the atherosclerosis component.

AoC initiation was associated with higher odds of prolonged post-initiation stay (OR, 2.325), and pre-hemodialysis vascular access with lower odds (OR, 0.205). The associations of GNRI and CRP remained after adjustment for these clinical background factors, suggesting that both markers may reflect aspects of disease burden during the hemodialysis initiation period that are not explained by these factors.

Changes in hemodialysis initiation and discharge practices over the two-decade study period may not be fully captured by adjustment for calendar period and may leave residual confounding. In the analysis restricted to 2014–2024, lnCRP remained associated with prolonged stay, whereas the GNRI estimate was attenuated and imprecise, with a confidence interval including both the full-cohort estimate and the null (Supplementary Table S2). The smaller sample may partly explain this imprecision, but a weaker GNRI association in more recent patients cannot be excluded.

The interpretation of the 30-day threshold warrants comment. Length of stay differed substantially by initiation modality and by preparedness for hemodialysis, so this threshold may preferentially capture patients with AoC initiation or without pre-hemodialysis vascular access. However, the associations were directionally consistent across alternative cutoffs, persisted in continuous, count, and time-to-event models that do not require dichotomization, and were observed within the stratum of patients with pre-hemodialysis vascular access, suggesting that the overall pattern was not unique to the 30-day threshold.

No uniform GNRI threshold has been established for patients initiating hemodialysis. The first quartile in our cohort (81.9) is somewhat lower than, but broadly comparable to, the lowest quartile (<84.9) reported by Takahashi et al. [13] using the same GNRI formula in incident hemodialysis patients. Analyses using previously reported literature-based thresholds (GNRI <92; CRP >3 mg/L) yielded results that were directionally consistent with those based on within-cohort quartiles, with adjusted odds ratios of 3.781 and 3.180, respectively, for patients with both features.

This study has several limitations. First, as a single-center retrospective study, residual confounding cannot be excluded; in particular, functional status and activities of daily living, which are established determinants of length of stay, were not recorded. Second, planned versus unplanned initiation was not documented and was not reconstructed retrospectively because classification would have depended on subjective judgment; we instead used pre-hemodialysis vascular access as an objective proxy for preparedness for hemodialysis. This proxy was based on access creation or catheter placement dates without uniform information on access maturity, and residual confounding by acute illness severity and unmeasured aspects of preparedness remains possible. Third, admission dates were not recorded, so the interval from admission to initiation, the total length of hospitalization, and the reason for admission could not be evaluated. Fourth, session-level hemodialysis prescriptions were not recorded, although initiation prescriptions generally followed an institutional approach, and CRP was available only at initiation in our database, precluding assessment of how inflammation evolves during the first weeks of hemodialysis. Fifth, the phenotype cutoffs were derived from within-cohort quartiles and have not been externally validated, so estimates based on these thresholds may be subject to overfitting. Finally, exclusion of patients with missing data may have introduced selection bias, and the primary outcome reflects hospitalization practices specific to Japan and may not be directly generalizable to other settings.

In conclusion, among patients who initiated maintenance hemodialysis during hospitalization, lower GNRI and higher CRP immediately before the first session were independently associated with prolonged post-initiation stay, and patients with both features showed the strongest association. This pattern should be interpreted as a joint association with a complicated initiation course rather than as evidence of biological synergy, and predictive performance beyond serum albumin was not demonstrated. Prospective multicenter studies are needed to determine whether assessment of nutritional risk and inflammation at hemodialysis initiation can inform care during this period.

## Supplementary Material

Supplementary_Materials_20261008.docx

## Data Availability

The data that support the findings of this study are available on request from the corresponding author, H.U. The data are not publicly available because they contain information that could compromise participant privacy and are subject to institutional restrictions imposed by the Ethics Committee of Toho University School of Medicine.
