# Supplementary material for: Nutritional risk, inflammation, and hospitalization after hemodialysis initiation
Source: Ren Fail. 2026 Nov 11;48(1):2757931. doi: 10.1080/0886022X.2026.2757931 (PMC13757912; doi:10.1080/0886022X.2026.2757931)
Supplement: Supplementary file 1 [file IRNF_A_2757931_SM2969.docx]

**Supplementary Materials**

*Nutritional Risk, Inflammation, and Hospitalization After Hemodialysis Initiation*

Unless otherwise specified, multivariable models were adjusted for age, sex, history of cardiovascular disease, diabetes mellitus, initiation modality (chronic vs. acute-on-chronic), calendar period, and pre-hemodialysis vascular access status, where applicable. GNRI and lnCRP were entered simultaneously in the same model, except in Supplementary Table S1. Robust standard errors were used. The outcome was prolonged post-initiation stay (≥30 days) unless otherwise indicated. For analyses treating GNRI and lnCRP as continuous variables, effect estimates are expressed per 1-unit increase. CI, confidence interval; CRP, C-reactive protein; GLM, generalized linear model; GNRI, Geriatric Nutritional Risk Index; HR, hazard ratio; IRR, incidence rate ratio; lnCRP, natural logarithm of (CRP + 0.1), with CRP expressed in mg/L; LOS, length of stay; OR, odds ratio; SHR, subdistribution hazard ratio; WBC, white blood cell count.

**Supplementary Table S1.**

**Exploratory analyses based on malnutrition, inflammation, and atherosclerosis components (n = 501)**

| **Group** | **n** | **Adjusted OR (95% CI)** | **P value** |
| --- | --- | --- | --- |
| **Panel A. MIA score (malnutrition + inflammation + atherosclerosis)** | | | |
| Score 0 (reference) | 66 | 1.00 (reference) | — |
| Score 1 | 148 | 1.273 (0.503–3.219) | 0.610 |
| Score 2 | 200 | 2.838 (1.143–7.043) | 0.025 |
| Score 3 | 87 | 3.835 (1.401–10.494) | 0.009 |
| Per 1-point increase (continuous) | 501 | 1.680 (1.257–2.246) | <0.001 |
| **Panel B. Malnutrition–inflammation (M+I) score, atherosclerosis component removed** | | | |
| Score 0 (reference) | 106 | 1.00 (reference) | — |
| Score 1 | 197 | 1.225 (0.587–2.556) | 0.589 |
| Score 2 | 198 | 3.781 (1.807–7.913) | <0.001 |
| **Panel C. Eight phenotype groups defined by individual components** | | | |
| M− I− A− (reference) | 66 | 1.00 (reference) | — |
| M− I− A+ | 40 | 1.328 (0.391–4.516) | 0.649 |
| M− I+ A− | 32 | 0.769 (0.217–2.720) | 0.684 |
| M− I+ A+ | 22 | 2.184 (0.497–9.606) | 0.301 |
| M+ I− A− | 76 | 1.592 (0.577–4.392) | 0.369 |
| M+ I− A+ | 67 | 1.258 (0.431–3.669) | 0.675 |
| M+ I+ A− | 111 | 4.522 (1.747–11.707) | 0.002 |
| M+ I+ A+ | 87 | 3.921 (1.417–10.856) | 0.009 |

Malnutrition (M) was defined as GNRI <92 and inflammation (I) as CRP >3 mg/L; atherosclerosis (A) was approximated by a history of cardiovascular disease. To maintain a consistent adjustment set across panels, history of cardiovascular disease was not included as a covariate in any panel, because it was used to define the atherosclerosis component in Panels A and C. In these models, the MIA score, malnutrition–inflammation score, or phenotype groups were entered in place of GNRI and lnCRP, with score 0 or M− I− A− as the reference.

**Supplementary Table S2.**

**Stratified and restricted analyses**

| **Analysis** | **n** | **GNRI OR (95% CI)** | **P value** | **lnCRP OR (95% CI)** | **P value** |
| --- | --- | --- | --- | --- | --- |
| **Multivariable primary analysis** | **501** | **0.955 (0.925–0.986)** | **0.005** | **1.203 (1.055–1.372)** | **0.006** |
| **By pre-hemodialysis vascular access status** | | | | | |
| Pre-hemodialysis vascular access present | 383 | 0.961 (0.924–0.998) | 0.042 | 1.255 (1.071–1.471) | 0.005 |
| Pre-hemodialysis vascular access absent | 118 | 0.931 (0.878–0.986) | 0.015 | 1.065 (0.840–1.351) | 0.604 |
| **Restricted cohorts** | | | | | |
| Chronic initiation only | 437 | 0.962 (0.931–0.995) | 0.024 | 1.195 (1.035–1.379) | 0.015 |
| Initiation in 2014–2024 | 263 | 0.978 (0.932–1.027) | 0.369 | 1.378 (1.103–1.723) | 0.005 |
| Survived to discharge | 493 | 0.956 (0.925–0.988) | 0.007 | 1.204 (1.054–1.376) | 0.006 |
| **By GNRI weight-component capping status** | | | | | |
| Weight ratio not capped | 174 | 0.918 (0.871–0.968) | 0.001 | 1.131 (0.922–1.389) | 0.238 |
| Weight ratio capped at 1 | 327 | 0.977 (0.937–1.020) | 0.291 | 1.259 (1.053–1.505) | 0.011 |

Models stratified or restricted by pre-hemodialysis vascular access status or initiation modality did not include the corresponding variable as a covariate. Interaction P values for pre-hemodialysis vascular access status were 0.304 for GNRI and 0.408 for lnCRP. For the four-phenotype analysis, low GNRI was defined as the lowest quartile of GNRI (≤81.9) and high CRP as the highest quartile of CRP (>13.9 mg/L) within the main analysis cohort. Among patients with pre-hemodialysis vascular access, adjusted ORs for the GNRI/CRP phenotypes (reference: neither low GNRI nor high CRP) were 1.626 (95% CI 0.728–3.630; P = 0.236) for low GNRI only, 2.177 (0.897–5.280; P = 0.085) for high CRP only, and 4.757 (1.803–12.554; P = 0.002) for low GNRI with high CRP. Capping refers to truncation of the actual-to-ideal body-weight ratio at 1 in the GNRI formula. Analyses by capping status were exploratory; interaction by capping status was not formally tested.

**Supplementary Table S3.**

**Alternative length-of-stay cutoffs defining prolonged post-initiation stay**

| **Cutoff** | **Events** | **GNRI OR (95% CI)** | **P value** | **lnCRP OR (95% CI)** | **P value** |
| --- | --- | --- | --- | --- | --- |
| ≥14 days | 368 | 0.945 (0.915–0.975) | <0.001 | 1.149 (1.019–1.296) | 0.024 |
| ≥21 days | 258 | 0.972 (0.944–1.000) | 0.053 | 1.176 (1.047–1.322) | 0.006 |
| **≥30 days (primary)** | **166** | **0.955 (0.925–0.986)** | **0.005** | **1.203 (1.055–1.372)** | **0.006** |
| ≥34 days (75th percentile) | 131 | 0.945 (0.913–0.979) | 0.002 | 1.120 (0.975–1.287) | 0.110 |
| ≥45 days | 82 | 0.955 (0.917–0.994) | 0.025 | 1.220 (1.034–1.440) | 0.019 |
| ≥60 days | 51 | 0.947 (0.903–0.993) | 0.026 | 1.262 (1.030–1.546) | 0.025 |

Event counts shown are for the main analysis cohort (n = 517); all multivariable ORs were estimated in the 501-patient complete-covariate cohort. The 34-day cutoff corresponds to the 75th percentile of post-initiation length of stay in the main analysis cohort.

**Supplementary Table S4.**

**Alternative outcome definitions and modeling strategies (n = 501)**

| **Model** | **Outcome / effect measure** | **GNRI (95% CI)** | **P value** | **lnCRP (95% CI)** | **P value** |
| --- | --- | --- | --- | --- | --- |
| **Logistic regression** | **≥30 days; OR** | **0.955 (0.925–0.986)** | **0.005** | **1.203 (1.055–1.372)** | **0.006** |
| Logistic regression | ≥30 days or in-hospital death; OR | 0.954 (0.924–0.985) | 0.004 | 1.218 (1.068–1.389) | 0.003 |
| Linear regression | ln(LOS); β | −0.016 (−0.024 to −0.009) | <0.001 | 0.051 (0.020–0.082) | 0.001 |
| Negative binomial | LOS in days; IRR | 0.979 (0.968–0.990) | <0.001 | 1.072 (1.036–1.109) | <0.001 |
| Gamma GLM, log link | LOS in days; mean ratio | 0.979 (0.968–0.990) | <0.001 | 1.073 (1.037–1.109) | <0.001 |
| Cox regression | Time to discharge alive; HR | 1.027 (1.013–1.041) | <0.001 | 0.897 (0.857–0.940) | <0.001 |
| Fine–Gray regression | Time to discharge alive; SHR | 1.025 (1.011–1.039) | <0.001 | 0.890 (0.850–0.932) | <0.001 |

For the Cox and Fine–Gray models, discharge alive was the event of interest (493 events); in-hospital deaths were censored at the time of death in the Cox model and treated as competing events in the Fine–Gray model. For time-to-discharge models, HR or SHR >1 indicates earlier discharge.

**Supplementary Table S5.**

**Additional adjustments**

| **Model** | **n** | **GNRI OR (95% CI)** | **P value** | **lnCRP OR (95% CI)** | **P value** |
| --- | --- | --- | --- | --- | --- |
| **Panel A. Adjustment for white blood cell count** | | | | | |
| **Without WBC adjustment** | **500** | **0.955 (0.925–0.986)** | **0.005** | **1.201 (1.054–1.370)** | **0.006** |
| + WBC, continuous | 500 | 0.956 (0.926–0.987) | 0.006 | 1.136 (0.989–1.304) | 0.072 |
| + WBC ≥10,000/µL, binary | 500 | 0.957 (0.926–0.989) | 0.008 | 1.140 (0.996–1.305) | 0.058 |
| **Panel B. Other additional adjustments** | | | | | |
| + Pulmonary edema | 487 | 0.957 (0.927–0.989) | 0.008 | 1.180 (1.033–1.348) | 0.015 |
| + Malignancy, autoimmune disease, and respiratory disease | 500 | 0.956 (0.926–0.987) | 0.006 | 1.198 (1.049–1.368) | 0.008 |
| + Primary kidney disease | 501 | 0.952 (0.921–0.984) | 0.003 | 1.209 (1.055–1.386) | 0.006 |

Panel A was restricted to the 500 patients with available WBC data, and sample sizes in Panel B vary according to the availability of each added covariate. Primary kidney disease was entered as the six categories shown in Supplementary Table S6; patients without a recorded primary kidney disease (n = 11) were included in the "Unknown or not recorded" category.
**Supplementary Table S6.**

**Initiation modality, vascular access status, and primary kidney disease by calendar period**

| **Variable** | **2004–2008** | **2009–2013** | **2014–2018** | **2019–2024** | **P value** |
| --- | --- | --- | --- | --- | --- |
| Patients, n | 118 | 135 | 134 | 130 | — |
| Acute-on-chronic initiation, n (%) | 29 (24.6) | 27 (20.0) | 9 (6.7) | 3 (2.3) | <0.001 |
| Pre-hemodialysis vascular access, n/N (%) | 73/103 (70.9) | 112/135 (83.0) | 96/133 (72.2) | 102/130 (78.5) | 0.086 |
| **Primary kidney disease, n (%)** | | | | | 0.058 |
| Diabetic nephropathy | 59 (50.0) | 58 (43.0) | 54 (40.3) | 61 (46.9) |  |
| Chronic glomerulonephritis | 13 (11.0) | 24 (17.8) | 28 (20.9) | 26 (20.0) |  |
| Nephrosclerosis | 7 (5.9) | 20 (14.8) | 17 (12.7) | 10 (7.7) |  |
| Polycystic kidney disease | 2 (1.7) | 1 (0.7) | 2 (1.5) | 6 (4.6) |  |
| Other | 16 (13.6) | 12 (8.9) | 8 (6.0) | 7 (5.4) |  |
| Unknown or not recorded | 21 (17.8) | 20 (14.8) | 25 (18.7) | 20 (15.4) |  |

P values were obtained using the chi-square test. The P value for primary kidney disease compares the distribution across all six categories. The category "Unknown or not recorded" includes 11 patients with no recorded primary kidney disease.
